# Problem gambling among people with first-episode psychosis: protocol for a prospective multicenter cohort study

**DOI:** 10.1186/s12888-023-04741-9

**Published:** 2023-04-25

**Authors:** Olivier Corbeil, Manuel Soulard, Maxime Huot-Lavoie, Laurent Béchard, Émilien Fournier, Sébastien Brodeur, Anne-Marie Essiambre, Charles Desmeules, Chantale Thériault, Amal Abdel-Baki, Christian Jacques, Isabelle Giroux, Michel Dorval, Marc-André Roy, Marie-France Demers

**Affiliations:** 1 Faculty of Pharmacy, Université Laval, Av. de la Médecine Quebec City (QC), Québec, 1050, G1V 0A6 Canada; 2 Quebec Mental Health University Institute, Québec, Canada; 3 CERVO Brain Research Centre, Québec, Canada; 4 Faculty of Medicine, Department of Psychiatry, Université Laval, Québec, Canada; 5 School of Psychology, Université Laval, Québec, Canada; 6 Faculty of Medicine, Department of Psychiatry, Université de Montréal, Québec, Canada; 7 Montreal University Hospital Research Center, Québec, Canada; 8 Centre Québécois d’Excellence pour la Prévention et le Traitement du Jeu, Québec, Canada; 9 CHU de Québec-Université Laval Research Center, Québec, Canada

**Keywords:** Psychotic disorder, Schizophrenia, First-episode psychosis, Gambling, Problem gambling, Gambling disorder, Comorbidity, Cohort, Multicenter

## Abstract

**Background:**

The limited available data suggest that the prevalence of problem gambling is increased among young adults with first-episode psychosis, possibly due in part to several risk factors for problem gambling that are common in this population. Aripiprazole, a widely used antipsychotic drug, has also been linked to cases of problem gambling, but causality remains uncertain. Although the consequences of problem gambling further hinder the recovery of people with first-episode psychosis, there is a paucity of research about this comorbidity and its risk factors. Additionally, to our knowledge, no screening instrument for problem gambling tailored to these individuals exists, contributing to its under-recognition. Further, treatment approaches for problem gambling adapted to this population are at an embryonic stage, while existing treatments effectiveness remains to be documented. Using an innovative screening and assessment procedure for problem gambling, this study aims to identify risk factors for problem gambling among people with first-episode psychosis and to document the effectiveness of standard treatment approaches.

**Methods:**

This is a multicenter prospective cohort study conducted in two first-episode psychosis clinics, including all patients admitted between November 1st, 2019, and November 1st, 2023, followed for up to 3 years until May 1st, 2024. These 2 clinics admit approximately 200 patients annually, for an expected sample size of 800 individuals. The primary outcome is the occurrence of a DSM-5 diagnosis of gambling disorder. All patients are screened and evaluated for problem gambling using a systematic procedure at admission, and every 6 months thereafter. Socio-demographic and clinical variables are prospectively extracted from the patients’ medical records. The nature and effectiveness of treatments for problem gambling offered to affected individuals are also documented from medical records. Survival analyses with Cox regression models will be used to identify potential risk factors for problem gambling. Descriptive statistics will document the effectiveness of treatments for problem gambling in this population.

**Discussion:**

A better understanding of potential risk factors for problem gambling among people with first-episode psychosis will allow for better prevention and detection of this neglected comorbidity. Results of this study will also hopefully raise clinicians’ and researchers’ awareness and serve as the basis to adapted treatments that will better support recovery.

**Trial registration:**

ClinicalTrials.gov, NCT05686772. Retrospectively registered, 9 January 2023.

## Background

There is no denying that gambling is a popular activity, with about 2 in 3 adults reporting some type of gambling in 2018, in Canada [1]. While most of these people only gamble occasionally, 0.12–5.8% of the global population has problem gambling (PBG) [2], which can be defined as a gambling behavior that causes harmful consequences for the person, his social network and community [3]. PBG frequently co-occurs with other mental health disorders, including mood and anxiety disorders, as well as substance use and personality disorders [4]. There is also evidence to suggest that the prevalence of PBG may be up to 4 times higher in people with psychotic disorders compared to the general population [5–7]. This proportion seems to be even greater among young people with first-episode psychosis (FEP), as suggested by the only study to date examining PBG among this population [8]. In this study, the prevalence of PBG of 6.4% among 219 patients was found to be 16 times higher than the 0.4% found in the general population [9]. In addition to a possible sharing of genetic influences between psychotic disorders and PBG [10], this comorbidity may also be due in part to several risk factors for PBG that are common among people with FEP, including an over-representation of males, young age, frequent psychiatric comorbidities, such as substance use disorders and personality disorders, as well as low socioeconomic status and homelessness [11]. While these risk factors have mostly been documented in general population samples, there is a paucity of data specific to individuals with psychotic disorders. Furthermore, there also appeared to be an association between the occurrence of PBG and aripiprazole, a widely used antipsychotic drug for the treatment of FEP, which had already been reported in previous case reports [12, 13]. Indeed, among the 14 FEP patients who developed PBG, 12 cases occurred during aripiprazole treatment, resulting in an adjusted odds ratio of 8.6 (*p*-value = 0.012). However, the retrospective design of this study, preventing the assessment of a potential causal link, and the limited existing literature do not allow firm conclusions to be drawn about this possible association [8]. Furthermore, there was no systematic screening for PBG at the study site at that time, which may have led to detection and monitoring bias.

The consequences of PBG are manifold and include financial hardship, broken social relationships and isolation, psychological distress and an increased risk of suicide [14, 15]. Although data regarding the consequences of PBG among individuals with a psychotic disorder are scarce, these can certainly be hypothesized as being all the more amplified [16–18]. Indeed, psychotic disorders are associated with several repercussions that can be exacerbated by the concomitant presence of PBG, including an increased rate of suicide and violent acts, stigma, low employment rates, and a 15-to-20-year decrease in life expectancy [19–21]. Not surprisingly, treatment of psychiatric comorbidities, which are common in people with a psychotic disorder, is critical to recovery [22, 23]. Despite this, to our knowledge, there are no tools to screen for PBG specifically tailored to this population, while approaches to treating PBG comorbid to a psychotic disorder are virtually non-existent [24–26]. Findings generated by these unmet needs is a low rate of PBG screening among people with a psychotic disorder by healthcare professionals and suboptimal treatment, hindering their hope for recovery [27–30].

Using an innovative screening and assessment procedure for PBG tailored to young adults with FEP, the main aim of this study is to test the hypothesis that risk factors for PBG in this population include substance use disorders, personality disorders, and the use of aripiprazole or other antipsychotics sharing the same mechanism of action (i.e., partial dopamine agonism). It also aims to demonstrate that current treatments for PBG, which have not been adapted for people with FEP, are currently insufficient to lead to recovery.

## Methods

### Study design

This is a prospective multicenter cohort study with an expected sample size of 800 patients with a diagnosis of FEP, followed for up to 3 years. The recruitment is ongoing since November 1st, 2019, through November 1st, 2023. All patients admitted at the 2 study sites during this period will be evaluated for PBG by the clinical staff at admission, and every 6 months thereafter, using a screening and assessment procedure developed specifically for this study. The follow-up period will end on May 1st, 2024. During this follow-up, independent variables will be extracted from patients’ medical records by the research staff at admission, and every 6 months afterwards.

### Study settings

This study is conducted in 2 FEP programs in the province of Quebec, Canada. These multidisciplinary clinics admit approximately 200 patients annually, who are followed for up to 3 years on a case management basis. In this clinical model, which is based on a Quebec adaptation of the NICE guidelines for FEP programs [31, 32], each patient is followed by a psychiatrist and a case manager, i.e., the clinician who oversees and coordinates the patient’s care and services. Case managers include trained nurses, occupational therapists, psychologists, social workers and specialized educators. In addition to pharmacological treatments, for which access to pharmacists specialized in mental health is provided, individual psychotherapy, family intervention and community outreach are also offered to patients during their follow-up. An exhaustive systematic clinical follow-up is provided by the case managers, which includes standardized questionnaires and also calls on the input of the patients’ relatives. All these clinical data are rigorously recorded in the patients’ medical files, and then collected and managed by the research staff using the Research Electronic Data Capture (REDCap) platform hosted at Université Laval [33, 34]. No consent to participate in this study is required given the lack of any contact between the patients and the research team (ethics approval obtained at both study sites, #MP-13-2020-1843, NSM).

### Participants

All patients admitted at the 2 study sites during the recruitment period will be included, without any exclusion criteria other than those related to admittance in the clinics (i.e., organic psychosis and severe intellectual disability). Admittance in these clinics requires to be aged between 18 and 35 years old, to have a primary diagnosis of FEP according to DSM-5 criteria (including both affective and nonaffective psychoses) and to have a limited exposure to antipsychotic continuous treatment (i.e., fewer than or equal to 6 months).

### Dependent variables

The primary outcome is the occurrence of a DSM-5 diagnosis of gambling disorder established by the treating psychiatrist. The secondary outcome is the occurrence of PBG, as defined by a score greater than or equal to 8 on the Problem Gambling Severity Index (PGSI) [3], a reliable and validated screening instrument in general population samples which has also been used in previous studies conducted in patients with psychotic disorders [5, 7, 8]. As previously stated, these outcomes are assessed by the case managers and treating psychiatrists using a screening procedure for PBG that has been developed in collaboration with experts on gambling from the *Centre Québécois d’Excellence pour la Prévention et le Traitement du Jeu*. It is a 3-step procedure that includes (1) a screening questionnaire of gambling habits, (2) an evaluation of the severity of the gambling problematic using the PGSI, and (3) a diagnostic interview assessing the DSM-5 gambling disorder diagnosis. The first 2 steps are carried out by the patients’ case managers, while the last step is conducted by the treating psychiatrist on the basis of all available data, i.e., his or her own clinical interview, information obtained during the screening procedure, and collateral information gathered by the case manager from the patients’ relatives. Results of this procedure are recorded in the patients’ medical files and extracted by the research staff.

### Independent variables

Variables that will be prospectively extracted from the patients’ medical records include socio-demographic (sex at birth, gender identity, ethnicity, employment status, education level, relationship status, living arrangements, criminal history) and clinical variables (main DSM-5 psychiatric diagnosis, psychotic illness severity as rated by the treating psychiatrist using the Clinical Global Impressions Severity scale, comorbid DSM-5 psychiatric diagnoses, community treatment order, medication treatment, including antipsychotics and all other psychotropic drugs [i.e., antidepressants, benzodiazepines, hypnotics, mood stabilizers, psychostimulants]). Regarding medication, information on both current (i.e., during the follow-up period) and past (i.e., prior to admission in the clinics) drugs will be collected (including dosages, frequency, and route of administration). A patient will be considered exposed to a drug after receiving it for greater than 1 month (or 1 injection in the case of long-acting injectable antipsychotics). Medications administered on an as-needed basis will not be considered. Gambling history (i.e., prior to patients’ admission in the clinics) will be questioned by the case managers as part of the screening procedure for PBG and divided in 3 categories, i.e., none, occasional/recreational, or PBG (i.e., DSM-3 or DSM-4 diagnosis of pathological gambling or DSM-5 diagnosis of gambling disorder). For patients with PBG and/or gambling disorder, the nature of the treatments received for their gambling problematic as well as their effectiveness in resolving it will be documented.

### Statistical analyses

Patients’ socio-demographic and clinical characteristics will be detailed using descriptive statistics. Survival analyses using Cox regression models will be performed to identify potential risk factors for both primary and secondary outcomes. Potential risk factors that will be examined include sex at birth, gender identity, ethnicity, employment and relationship status, criminal history, psychiatric comorbidities (i.e., substance-use disorder, personality disorder), the use of aripiprazole, the use of dopamine partial agonists other than aripiprazole (i.e., brexpiprazole, cariprazine) and the use of any dopamine partial agonists. Cox regression models will be constructed for each of these variables to adjust for potential confounding factors identified using the directed acyclic graph approach. In order to detect a hazard ratio greater than 4 between the use of aripiprazole and the primary outcome with a statistical power of 90% and a bilateral significance level of 5%, 25 events are needed through the follow-up period. The hazard ratio of 4 is considered conservative considering that an odds ratio of 8.6 was observed in our previous nested case-control study [8]. The expected sample size of 800 patients is considered sufficient to observe at least 25 cases of gambling disorder (primary outcome) over the follow-up period given the 3-year prevalence of PBG of 6.4% previously revealed in a similar population [8]. As for the nature and the effectiveness of treatments for PBG and/or gambling disorder, this will be documented for all patients who will have developed either the primary or secondary outcome during the study follow-up period using descriptive statistics.

## Discussion

This prospective multicenter cohort study aims to identify potential risk factors for PBG in young adults with FEP. Among these factors, the causality of the link between the use of aripiprazole, an antipsychotic drug, and PBG, remains to be better documented, to which this prospective study will greatly contribute. Although the comorbidity of PBG and psychotic disorders has been neglected so far, it does nevertheless significantly hinder recovery of the affected individuals. For this reason, results of this study will be crucial to clinicians providing care to people with FEP. The knowledge of potential risk factors will allow them to better prevent and detect the occurrence of PBG. For instance, a better understanding of the possible association between the use of aripiprazole and PBG might ultimately influence the treatment choice through a shared-decision process, even more so in individuals at increased risk for PBG. In addition, to our knowledge, there is no screening instrument for PBG that has been specifically developed for people with FEP, which further contributes to the underestimation of this comorbidity. To this end, the procedure created for this study, which will further be refined following its completion, is expected to be spread out to all FEP programs in the province of Quebec and possibly across Canada. Such a systematic tool, in addition to information provided by the patients’ relatives, is essential for better, and earlier, detection of PBG. Treatment approaches for PBG tailored to people with psychotic disorders are still only at an embryonic phase. Thus, individuals presenting with this comorbidity are usually addressed to standard treatments that have been developed and tested for the general population. While these approaches can be hypothesized as suboptimal for people with FEP, this remains to be documented. Results of this study will hopefully raise the awareness of clinicians and researchers working in this field and serve as the basis to adapted treatments that will better support recovery.

The major strengths of this study include the large sample size and the length of the follow-up period, which should enable us to generate relevant findings. The population study is also representative of the FEP population of the province of Quebec, in part due to the lack of exclusion criteria and necessity to obtain consent as well as the territories served by the 2 study sites, which contain both urban and rural areas. Furthermore, the fact that no informed consent is required prevents a participation bias. Results of this study should thus be readily generalizable. In addition, the exhaustiveness and rigour of the screening and assessment procedure for PBG, as well as collateral information provided by the patients’ relatives, will ensure that the findings are reliable and less prone to detection bias. The prospective design of the study will also allow for a better examination of potential risk factors for PBG in this population and could add evidence to a possible causal link between aripiprazole use and PBG. To this end, the fact that all patients, notwithstanding the antipsychotic drugs received, will benefit from the same screening procedure for PBG will prevent a surveillance bias. The key limitations to this study are (1) previous findings regarding a possible association between PBG and aripiprazole could lead to a decrease in its use, particularly in patients deemed more at risk by clinicians, and thus introduce a prescribing bias, and (2) evaluation of the study outcomes is dependent on the cooperation of clinicians in following the PBG screening procedure. For the first point, while we have no control over the clinical practices and prescribing patterns of psychiatrists, the study will be extended if 25 cases of PBG have not been observed by the end of the recruitment period, i.e., November 1st, 2023. For the second point, a monitoring committee has been set up to closely and regularly follow up with clinicians to ensure that the screening procedure for PBG is being properly followed. Training in the use of this procedure is also provided to all new clinicians arriving during the study. Taken together, these methodological aspects will lead to results that should significantly deepen our understanding of a neglected comorbidity and hopefully positively impact services and care provided to people with FEP.

## Data Availability

Not applicable.

## Abbreviations

DSM: Diagnostic and Statistical Manual of Mental Disorders
FEP: First-episode psychosis
PBG: Problem gambling
PGSI: Problem Gambling Severity Index
REDCap: Research Electronic Data Capture.
